# Elastofibroma dorsi: imaging-guided minimally invasive treatment using percutaneous CO_2_ cryoablation

**DOI:** 10.1007/s00256-026-05302-3

**Published:** 2026-07-15

**Authors:** Giovanni Mauri, Pierluigi Glielmo, Francesco Cammarata, Domenico Albano, Luca Maria Sconfienza

**Affiliations:** 1 Dipartimento Di Scienze Biomediche Per La Salute, Università Degli Studi Di Milano, Via Festa del Perdono 7, 20123 Milan, Italy; 2 IRCCS Istituto Ortopedico Galeazzi, Via Cristina Belgioioso 173, 20157 Milan, Italy; 3 Dipartimento Di Scienze Biomediche, Chirurgiche Ed Odontoiatriche, Università Degli Studi Di Milano, Milan, Italy; 4 Dipartimento Di Radiologia, ASST Grande Ospedale Metropolitano Niguarda, Milan, Italy

**Keywords:** Elastofibroma dorsi, Cryoablation, Musculoskeletal intervention, Percutaneous treatment, Interventional radiology, MRI

## Abstract

**Objective:**

Elastofibroma dorsi is a benign slow-growing fibroelastic pseudotumoral lesion that typically arises in the infrascapular region of elderly individuals and is usually managed conservatively or by surgical excision when symptomatic. Percutaneous cryoablation has only recently been described for this entity, and experience remains very limited. We report the case of a symptomatic left elastofibroma dorsi treated by percutaneous cryoablation using a CO_2_-based system.

**Materials and methods:**

A 66-year-old man with non-painful but mechanically symptomatic left elastofibroma dorsi, unwilling to undergo surgery, came to our attention to be treated with minimally invasive approach. Pre-treatment MRI demonstrated a lesion measuring 48 × 18 × 70 mm (31.6 mL) in the typical subscapular location. The lesion was treated by US-CT-guided percutaneous cryoablation using a CO_2_-based system (Metrum Cryoflex, Lomianki, Poland) equipped with a 4.5 cm of exposed tip cryoapplicator.

**Results:**

The procedure was technically successful and well tolerated, with no immediate or delayed complications and free from immediate and post-treatment pain. At 3-month follow-up, contrast-enhanced MRI showed size reduction to 38 × 12 × 59 mm (14.1 mL, − 55.3% volume reduction), with internal non-enhancing necrotic change consistent with post-ablation effect. Clinically, the patient reported complete resolution of mechanical symptoms.

**Conclusions:**

This case suggests that percutaneous CO_2_ cryoablation may represent a feasible minimally invasive treatment option for selected patients with symptomatic elastofibroma dorsi. The use of a CO_2_-based cryoablation platform may offer practical and economic advantages, although its lower freezing capacity compared with conventional argon-and nitrogen-based systems requires careful procedural planning.

## Introduction

Elastofibroma dorsi is an uncommon benign fibroelastic pseudotumor that classically develops between the inferior scapula and the thoracic wall, usually deep to the serratus anterior and latissimus dorsi muscles [1, 2]. It predominantly affects older adults, especially women, and may be unilateral or bilateral. Although frequently incidental and asymptomatic, some patients present with infrascapular pain, stiffness, snapping scapula, discomfort during shoulder motion, or a palpable mass [3]. Its characteristic imaging appearance, especially on magnetic resonance imaging (MRI) and computed tomography (CT), is that of a poorly circumscribed soft-tissue lesion with internal alternating fibrous and fatty streaks in the typical infrascapular location [2, 3]. Ultrasound is particularly valuable for its dynamic properties, being able to show the mass popping out during upper limb motion [4].

Symptomatic lesions have traditionally been managed by surgical excision. Surgery is generally effective, but postoperative seroma, hematoma, delayed wound healing, and prolonged functional recovery are well-recognized concerns. In one modern surgical series of 70 cases, the authors specifically reported the non-negligible burden of postoperative complications, which accounted for 17% of cases [5, 6].

Image-guided cryoablation has emerged as a minimally invasive option for a wide spectrum of tumors as it allows accurate applicator placement, visualization of the ablation zone, and protection of adjacent structures with adjunctive maneuvers when needed [7–12]. Recent literature supports its safety and utility in selected soft-tissue tumors [7–9], but published experience in elastofibroma dorsi remains extremely limited.

A 2025 case report described successful percutaneous cryoablation of elastofibroma dorsi using a conventional argon-based system [13]. This system is however very expensive at our institution. CO_2_-based cryoablation systems are generally used for cryoneuromodulation of peripheral nerves to treat chronic pain. They cannot reach freezing temperature as low as argon- or nitrogen-based systems but are generally far cheaper.

In this technical report, we describe the use of a CO_2_-based cryoablation system for percutaneous treatment of elastofibroma dorsi.

## Material and methods

The patient provided written consent to perform the procedure and to publish his case in a scientific report.

A 66-year-old man, master swimming athlete, presented complaining of a symptomatic left infrascapular snapping and palpable swelling noted during swimming activity. At clinical examination, snapping and palpable mass were clearly noted during arm abduction and scapular elevation and had been present for about 5 months. During snapping, he never reported pain but only mild discomfort.

**Fig. 1:**
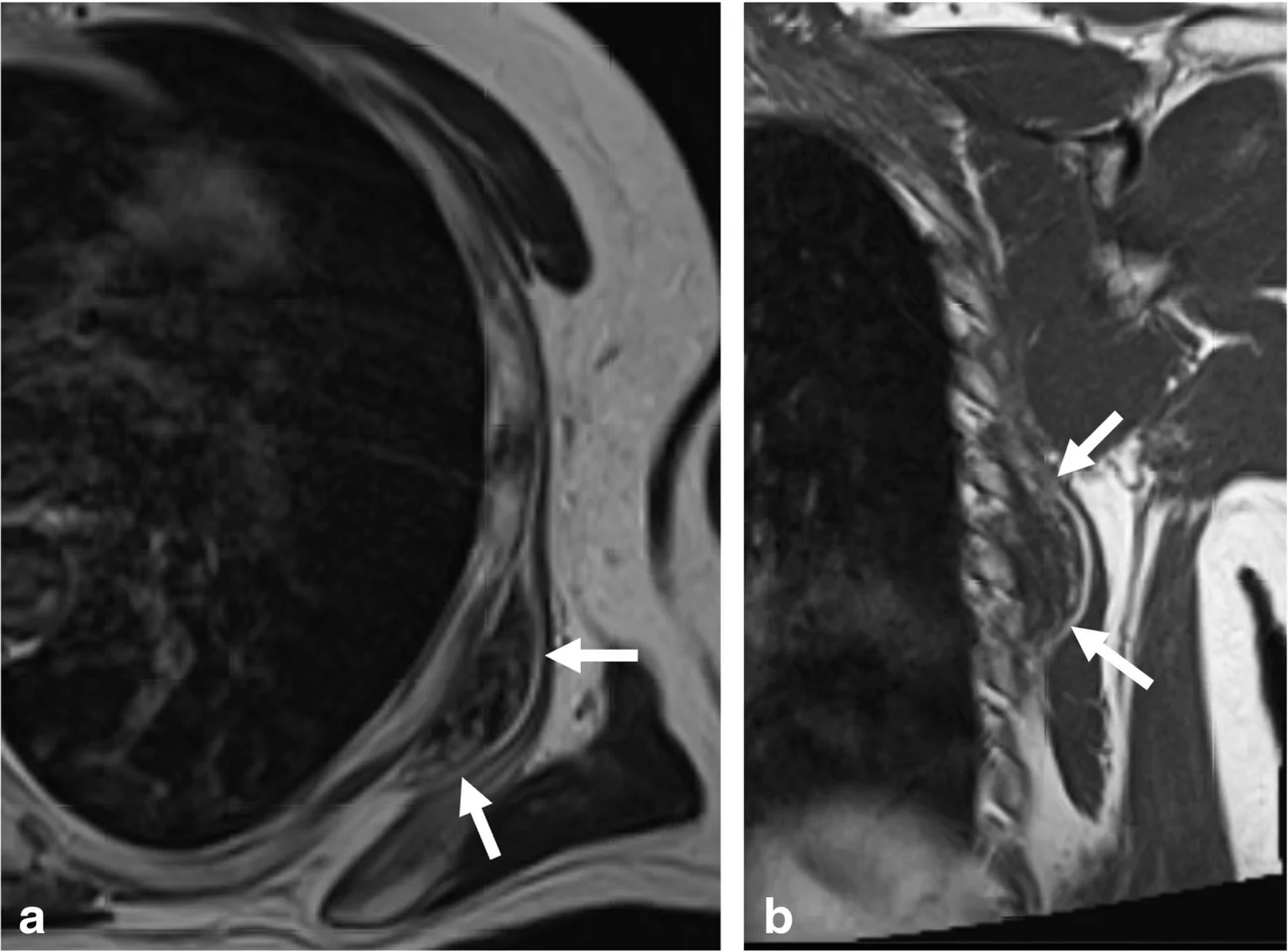
66-year-old man with symptomatic left elastofibroma dorsi. **a** Axial T2-weighted MR image (TE = 108 ms; TR = 4200 ms) shows a poorly circumscribed infrascapular soft-tissue mass with alternating fibrous low-signal and fatty high-signal streaks (arrows). **b** Coronal T1-weighted MR image (TE = 9.6 ms; TR = 375 ms) demonstrates the lesion interposed between the thoracic wall and adjacent scapulothoracic musculature (arrows)

Baseline 1.5 T MRI (Magnetom Altea, Siemens, Erlangen, Germany) demonstrated a lesion in the typical infrascapular location, interposed between the serratus anterior and thoracic wall, with the characteristic internal mix of fibrous and fatty components consistent with elastofibroma dorsi (Fig. 1) [1, 2]. No edema or signs of aggressive features were seen. No biopsy was performed due to the very typical imaging features. The lesion measured 48 × 18 × 70 mm (H × L × W × 0.523; volume = 31.6 mL). Despite the absence of clear pain, the patient wanted to remove the lesion as it was interfering with his sport activity. After surgical consultation, he autonomously decided to ask for interventional radiology evaluation as a potentially minimally invasive option. We thoroughly discussed the case with him, explaining clearly the potential advantages and limitations of this approach, including the choice of using a CO_2_ system rather than a conventional cryoablation system.

**Fig. 2:**
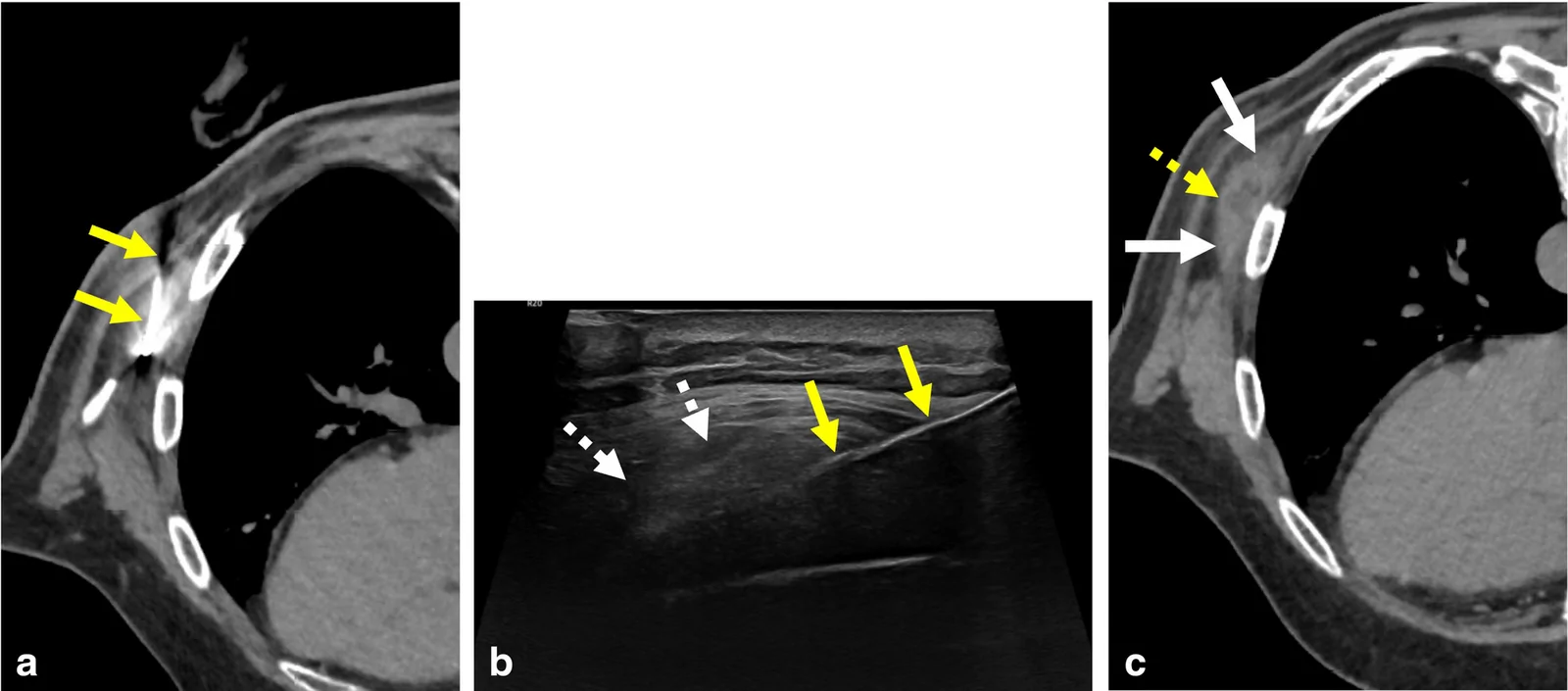
US and CT-guided percutaneous cryoablation of elastofibroma dorsi. **a** CT and **b** US image of the cryoapplicator inside the elastofibroma dorsi (yellow arrows). Note the ice ball (dotted white arrows) visible in the US image. **c** At the end of the procedure, non-contrast CT shows a necrotic area within the lesion (dotted yellow arrow)

The patient was positioned on the CT table (Revolution Ascend 128, General Electric, Milwaukee, USA) in almost-prone decubitus, with a support slightly elevating the left side of the chest. The procedure was performed under CT-US fusion guidance to combine the panoramic capabilities of CT with the dynamic use of US (Samsung Medison, Seoul, Korea) [14, 15]. The procedure was conducted under mild intravenous sedation (2 mL of midazolam chlorhydrate 5 mg/mL before the procedure, Midazolam B.Braun, B.Braun, Milan, Italy). Different from heat-based ablation techniques, freezing induces local analgesia itself, thus it is very well tolerated. A Metrum Cryoflex cryoablation system was used (Metrum Cryoflex, Lomianki, Poland) equipped with a 15-cm-long cryoapplicator with 4.5-cm active tip. This system relies on CO_2_ to generate the ice ball through the Joule-Thompson effect and reaches higher temperatures than conventional argon- or nitrogen-based cryoablation platforms. Perilesional local anesthesia (10 mL lidocaine chlorhydrate 2%, Monico, Mestre, Italy) and peri-lesional hydrodissection (warm 5% glucose solution, B.Braun, Milan, Italy) was performed to increase the distance of lesion from the surrounding structures [16–18]. One cryoapplicator was inserted into the more distal portion of lesion using ultrasound guidance and applicator position check was performed using CT. Cryoablation was then carried out using a cycle of 3-min freezing, 7-s active thawing, and 3-min freezing. Then, the applicator was retracted in a more proximal untreated area with a pull-back mechanism [16–18] and another freeze–thaw-freeze cycle was performed (Fig. 2). Passive thawing occurred during applicator repositioning. Intraprocedural ultrasound and CT monitoring demonstrated satisfactory coverage of the lesion during the ablation cycles. Procedure was terminated when imaging demonstrated a satisfactory ablation coverage of the area. Two freeze–thaw-freeze cycles were performed overall.

## Results

No immediate or delayed complications occurred. The procedure had a whole duration of 22 min from initial needle insertion to final CT check, and the post-procedural course was uneventful. The patient was kept in observation for two hours and then discharged, with the prescription of taking oral painkillers in case of pain and keeping the shoulder in motion but without any sport activity.

**Fig. 3:**
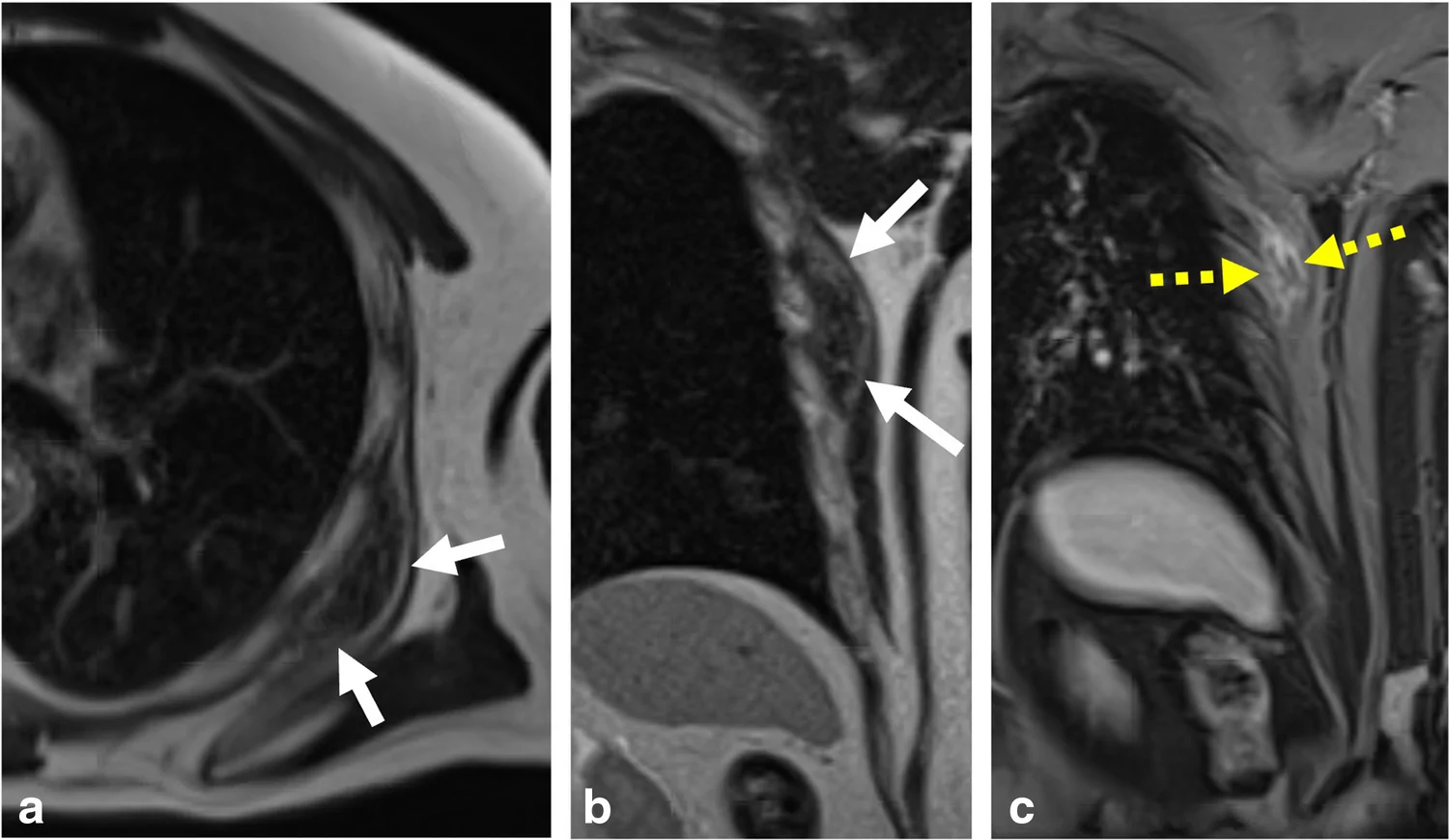
Three-month follow-up MRI after percutaneous cryoablation of elastofibroma dorsi. **a** Axial (TE = 98 ms; TR = 1400 ms) and **b** coronal (TE = 100 ms; TR = 1400 ms) HASTE T2-weighted MR images demonstrate interval size reduction of the treated lesion (white arrows). **c** Post-contrast coronal fat-suppressed 3D T1-weighted image (VIBE, TE = 2.39 ms; TR = 7.02 ms) shows internal non-enhancing necrotic change consistent with post-ablation effect (dotted yellow arrows)

After 3 months, the patient was again seen for follow-up. He subjectively reported total disappearance of discomfort and disappearance of the infrascapular snapping and palpable mass. Follow-up contrast-enhanced MRI demonstrated interval reduction in lesion size to 38 × 12 × 59 mm (volume = 14.1 mL, 55.3% volume reduction), together with internal non-enhancing necrotic geographic area after contrast administration, consistent with post-ablation effect (Fig. 3). No delayed complication such as pneumothorax, neuropathy, infection, or clinically relevant muscle injury was observed. The patient was able to resume his previous swimming activity.

## Discussion

Elastofibroma dorsi is a benign lesion in which treatment is generally reserved for symptomatic patients [1, 2]. Because the diagnosis can often be confidently suggested by imaging in the appropriate clinical setting, the main therapeutic goal is symptom relief rather than radical excision as it happens in malignant lesions [3, 4]. Surgery remains the standard treatment for symptomatic lesions, but its morbidity is not negligible, particularly in elderly patients or in those wishing to minimize convalescence and preserve shoulder function [3, 5].

Cryoablation offers several theoretical and practical advantages in this setting. The ablation zone can be monitored during treatment, and the technique may be combined with hydrodissection or pneumodissection when the target is close to vulnerable structures [6–8]. More broadly, contemporary musculoskeletal literature supports cryoablation as a safe and effective option for selected bone and soft-tissue tumors, with the caveat that complication rates vary according to lesion site, size, and adjacent anatomy [8, 9].

To date, however, the literature specific to elastofibroma dorsi is extremely sparse. The recent report by Mina et al. [13] appears to be the first published case of percutaneous cryoablation for elastofibroma dorsi and used a conventional argon-based platform. Our case differs in the use of a CO_2_-based low-power cryoablation system. This technical distinction is relevant because the available cryoablation modalities have all different features. Nitrogen-based systems reach a temperature as low as − 196 °C, but the setup of the system requires large nitrogen cylinders, which should be located outside the operation room and long pipes connecting to the applicator. Argon-based systems are definitely easier to set up and can reach nadir temperatures as low as 180 °C, with both modalities being excellent for ablation of malignant lesions. However, at our institution, those systems are quite expensive, as a single application cost ranges between Euro 3,000,00.- and Euro 5,000,00.-. On the other hand, CO_2_-based systems reach a higher nadir temperature (− 78.5 °C). Thus, this modality is not adequate in malignant lesions but, when the goal is debulking as in our case, a CO_2_-based system, together with meticulous applicator positioning and close imaging monitoring, may be sufficient to treat even a relatively fibrous lesion such as elastofibroma dorsi. This was our main concern of using this technique, which was clearly explained to the patient. Also, cost of a single application with a CO_2_-based system was around Euro 500,00.-.

In the present case, treatment was followed by both radiologic and clinical response. Follow-up MRI demonstrated interval reduction in lesion volume of about 55%, together with internal post-contrast non-enhancing necrotic change. While symptom improvement remains the most relevant endpoint in benign lesions, imaging findings provide objective corroboration of local treatment effect and suggest that even a lower-power cryotherapy platform may induce meaningful devascularization and tissue necrosis. Of note, we may speculate that MRI performed at a longer follow-up may show the reduction of the necrotic area and a further reduction of the mass. On the other hand, we have no data about the possible regrowth of the lesion, as some vital tissue is still in place. However, there is the theoretical possibility of a second percutaneous debulking ablation procedure, as it happens in other benign conditions [17, 18]. At the moment, we didn’t plan further imaging control, as patient’s symptoms were completely resolved. However, the patient was instructed to contact us again in case of symptom recurrence.

This report has several limitations. First, it describes a single case, so reproducibility, treatment indications and ideal patient selection cannot be defined. Second, the follow-up period was limited to 3 months, preventing conclusions regarding long-term durability or recurrence. Third, no direct comparison with surgery or with conventional argon- or nitrogen-based cryoablation or with other ablation modalities is possible. Finally, because elastofibroma dorsi is a benign lesion with often indolent behavior, clinical outcome should be considered first over imaging response.

In conclusion, this case supports the feasibility and safety of percutaneous low-power CO_2_-based cryoablation as a minimally invasive option for selected symptomatic elastofibroma dorsi. In patients who wish to avoid surgery, this technique may represent a reasonable lower-cost, easier-to-use alternative when the diagnosis is secure and adequate image-guided expertise is available.

## Data Availability

Not applicable.

## References

[1] Kudo S. Elastofibroma dorsi: CT and MR imaging findings. Semin Musculoskelet Radiol. 2001;5(2):103–5. 10.1055/s-2001-15661. 11500149

[2] Ochsner JE, Sewall SA, Brooks GN, Agni R. Best cases from the AFIP: elastofibroma dorsi. Radiographics. 2006;26(6):1873–6. 10.1148/rg.266055184. 17102057

[3] Muramatsu K, Ihara K, Hashimoto T, Seto S, Taguchi T. Elastofibroma dorsi: diagnosis and treatment. J Shoulder Elbow Surg. 2007;16(5):591–5. 10.1016/j.jse.2006.12.010. 17560807

[4] Aparisi Gómez MP, Errani C, Lalam R, et al. The role of ultrasound in the diagnosis of soft tissue tumors. Semin Musculoskelet Radiol. 2020;24(2):135–55. 10.1055/s-0039-3402060. 32438440

[5] Parratt MTR, Donaldson JR, Flanagan AM, et al. Elastofibroma dorsi: management, outcome and review of the literature. J Bone Joint Surg Br. 2010;92(2):262–6. 10.1302/0301-620X.92B2.22705. 20130320

[6] Scamporlino A, Ruggiero C, Aramini B, Morandi U, Stefani A. Surgery for elastofibroma dorsi: optimizing the management of a benign tumor—an analysis of 70 cases. J Thorac Dis. 2020;12(5):1884–94. 10.21037/jtd-20-649. 32642092 PMC7330361

[7] Papalexis N, Savarese LG, Peta G, et al. Role of percutaneous cryoablation in bone and soft tissue tumors. Curr Oncol. 2023;30(8):6744–70. 10.3390/curroncol30070495. 37504355 PMC10377811

[8] Bodard S, Marcelin C, Kastler A, et al. Safety and efficacy of cryoablation of soft-tissue tumours: a systematic review. Br J Radiol. 2025;98(1169):861–74. 10.1093/bjr/tqae075. 38588564 PMC12089779

[9] Orsi F, Hamiddin AS, Sattin C, et al. Liquid nitrogen-based cryoablation: complication rates for lung, bone, and soft tissue tumors cryoablation. Br J Radiol. 2024;97(1163):1863–9. 10.1093/bjr/tqae171. 39226178 PMC11491613

[10] Shyn PB, Mauri G, Alencar RO, et al. Percutaneous imaging-guided cryoablation of liver tumors: predicting local progression on 24-hour MRI. AJR Am J Roentgenol. 2014;203(2):W181–91. 10.2214/AJR.13.10747. 24555531

[11] Mauri G, Sconfienza LM, Pescatori LC, et al. Technical success, technique efficacy and complications of minimally-invasive imaging-guided percutaneous ablation procedures of breast cancer: a systematic review and meta-analysis. Eur Radiol. 2017;27(8):3199–210. 10.1007/s00330-016-4668-9. 28050693

[12] Mauri G, Nicosia L, Varano GM, et al. Unusual tumour ablations: report of difficult and interesting cases. Ecancermedicalscience. 2017;18(11):733. 10.3332/ecancer.2017.733. PMC5406223 28487751

[13] Mina D, Gustainyte V, Venkat S, Tong WL, Kocharyan H. Percutaneous cryoablation of elastofibroma dorsi in a 73-year-old woman: a novel minimally invasive alternative. Cureus. 2025;17(12):e99296. 10.7759/cureus.99296. 41542006 PMC12803006

[14] Carriero S, Della Pepa G, Monfardini L, et al. Role of fusion imaging in image-guided thermal ablations. Diagnostics (Basel). 2021;11(3):549. 10.3390/diagnostics11030549. 33808572 PMC8003372

[15] Mauri G, Gitto S, Pescatori LC, Albano D, Messina C, Sconfienza LM. Technical feasibility of electromagnetic US/CT fusion imaging and virtual navigation in the guidance of spine biopsies. Ultraschall Med. 2022;43(4):387–92. 10.1055/a-1194-4225. 32785900

[16] Mauri G, Nicosia L, Varano GM, et al. Tips and tricks for a safe and effective image-guided percutaneous renal tumour ablation. Insights Imaging. 2017;8(3):357–63. 10.1007/s13244-017-0555-4. 28500486 PMC5438321

[17] Mainini AP, Monaco C, Pescatori LC, et al. Image-guided thermal ablation of benign thyroid nodules. J Ultrasound. 2016;20(1):11–22. 10.1007/s40477-016-0221-6. 28298940 PMC5334266

[18] Mauri G, Nicosia L, Della Vigna P, et al. Percutaneous laser ablation for benign and malignant thyroid diseases. Ultrasonography. 2019;38(1):25–36. 10.14366/usg.18034. 30440161 PMC6323312

